# Consultation frequency patterns for older patients in Danish general practice: a nationwide register-based cohort study

**DOI:** 10.3399/BJGPO.2024.0234

**Published:** 2025-08-13

**Authors:** Jonas K Olsen, Sonja Wehberg, Frans Boch Waldorff, Daniel Pilsgaard Henriksen, Jesper Lykkegaard

**Affiliations:** 1 Research Unit for General Practice, Department of Public Health, University of Southern Denmark, Odense, Denmark; 2 Section of General Practice and The Research Unit for General Practice, Department of Public Health, University of Copenhagen, Copenhagen, Denmark; 3 Department of Clinical Pharmacology, Odense University Hospital, Odense, Denmark

**Keywords:** practice organisation, family medicine, care of older people, aged, general practice, cohort studies

## Abstract

**Background:**

There may be distinctly different ways for general practices to serve the growing population of older patients, providing them different combinations of face-to-face, telephone, and email consultations; home visits; and chronic care reviews.

**Aim:**

To identify latent general practice profiles of frequency and combination of consultation types for older patients and relate them to practice characteristics.

**Design & setting:**

Register-based cohort study of all Danish citizens aged ≥75 years.

**Method:**

For each of the years 2017–2021, a latent profile analysis was conducted on the practices’ frequencies of consultation types, adjusting for patient population characteristics.

**Results:**

We identified a ’Majority’ and the following three temporary latent profiles of provision of consultation services to older patients: the ’Phone heavy’ profile (9%–10% of practices, 2017–2019), which provided nearly double the telephone consultations as the ’Majority’ profile and was associated with the GPs being older and working single-handed; the ’High frequency’ profile (12%–14% of practices, 2017–2018), which provided higher levels of face-to-face, telephone, and email consultations than the ’Majority’ profile; and the ’Phone and email heavy’ profile (7% of practices, 2020), which provided more email than face-to-face consultations, and more of each consultation than the ’Majority’ profile. The number of profiles decreased from three in 2017 to only the ’Majority’ profile in 2021.

**Conclusion:**

There is a trend towards a more uniform pattern of consultations for older patients in general practice. It is unknown whether high provision of certain types of consultations in general practice has downstream effects such as decreased need for hospital and out-of-hours services.

## How this fits in

The worldwide shift in demographics towards an older population maybe the largest challenge for healthcare systems, especially for general practice. Our study shows that some practices service their older patients in a way that is markedly different from the majority. The alternative patterns of consultations involve more services, which from an isolated time and cost perspective must be a disadvantage in a tax-funded healthcare sector. However, if the increased level of consultations has positive downstream effects, such as decreased need for hospital and out-of-hours services, it may be better to use the allocated healthcare resources in daytime general practice than in the secondary services.

## Introduction

Older patients have the highest frequency of consultations in general practice.^1^ Worldwide, this age group is forecast to increase heavily, potentially overwhelming primary health care. Studies concerning older patients’ consultation frequencies and types in general practice have primarily been centred around one specific consultation type, such as telephone,^2^ email,^3^ or preventive home visits,^4^ if studied at all.^5^ While these studies provide valuable insights into the individual consultation modalities, there exists a notable gap in the exploration of how general practice provides combinations of different consultation types to the older population.

In Denmark, consultations in general practice can be divided into five types: face-to-face, telephone, email, home visits, and chronic care reviews. Chronic care reviews, which have seen increased focus in recent years,^6,7^ are provided primarily face-to-face at the surgery and less often as home visits. Consultations can be performed between the patient and the GP or other practice staff (for example, nurses) or between the GP and municipality services. To effectively serve the patients registered with a practice, adaptions must be made to local circumstances such as patient demands and distance to the nearest hospital. Therefore, variation among practices in consultation types and frequencies is expected. Systematic variation exceeding that of local circumstances could indicate problems with the quality of care or healthcare service delivery. However, our understanding of different types of consultations and how general practices choose to organise themselves to effectively serve their patients is limited.

Some consultations in general practice are initiated by the patient, others by the GP, such as follow-up on lab results and review consultations for chronic diseases. The frequency of consultations by email or telephone can be controlled by the GP; for example, by restricting the allocated time for answering phonecalls and emails, as well as using triage to control access to face-to-face, home visit, and chronic care reviews. This way to some extent the GP has the dominating say as to the frequency and type of consultations offered.

This study aims to identify latent general practice profiles of frequency and combination of consultation types for older patients and relate them to practice characteristics.

## Method

### Design, setting, and population

We conducted a nationwide register-based cohort study following a published protocol.^8^

The north-western European country of Denmark has approximately 5.8 million citizens. Most healthcare services, including in general practice, are fully tax funded. More than 98% of the population is registered with a self-chosen general practice. General practices are privately owned and paid by capitation fees (one-third) and fees for services (two-thirds).^9^ Older patients are on average registered with the same general practice for at least 9.5 years.^10^

For each of the years 2017–2021, the study population included all persons aged≥75 years who on their birthday in the given year were registered with a Danish general practice. All Danish general practices were included. However, practices with fewer than 500 registered or 20 eligible patients at the beginning or end of each study year were excluded in the current year. Practices without at least one remunerated consultation to a patient each month of the current year were excluded in the current year.

### Consultation types in Danish general practice

GPs electronically report each performed consultation to the regional health insurance for remuneration. The available consultations are regular consultation (attending the general practice), consultation by telephone or email, home visit, telephone or email consultation with municipality services, assessment of patients at nursing home, video conference with the municipality or secondary services, and talk therapy (Supplementary table S1). Only emails sent from the general practice are remunerated and thereby registered. Two main types of chronic care reviews exist: annual disease-specific chronic care review consultations; and preventive home visits. An annual chronic care review consultation can be remunerated once per patient per chronic condition per year. Preventive home visits can be performed once per year to frail older individuals (often aged ≥75 years with multiple chronic diseases).^11^ In addition, several regional special consultations (§2 services) and temporary services existed at the time of the study period during the COVID-19 pandemic (Supplementary table S1). Only consultation types performed ≥1000 times a year in the study population were included in the study.

### Statistical analysis

Data sources and linkage between sources has been described in detail in our protocol.^8^ To account for different patient populations in each general practice, we calculated an adjusted consultation frequency. The adjusted consultation frequency was calculated by dividing the crude consultation frequency in each general practice by a predicted consultation frequency based on their patient population and multiplying by the entire population’s average consultation frequency. The predicted consultation frequency was based on a zero-inflated Poisson regression, separately for each calendar year adjusting for patient health factors: age, sex, multimorbidity, ethnicity, polypharmacy, and level of home healthcare services; patient geographic factors: rural or urban district, travel distances from home address to the general practice and the local emergency department; and patient socioeconomic factors: cohabitation, household income, and household wealth. Definitions of adjustment variables is presented in Supplementary table S2. All analyses were made on the patient level. Separate analyses were made for each consultation type; that is, face-to-face, telephone, email, home visits, and chronic care reviews.

Using latent profile analysis,^12^ the practices were grouped according to the adjusted rates of face-to-face, telephone, email consultations, home visit, and chronic care reviews defining each practice’s pattern of consultations. We chose the highest number of profiles while ensuring at least 5% of the general practices within the smallest profile. We determined profile labels for each group based on differences on consultation frequencies and proportion of general practices. We used a multinomial logistic regression model to analyse practice factors (GP composition [number, age, sex, and seniority]) associated with latent profile with the largest latent profile as reference.

If GP data were missing, imputation based on the available GP data or, if none were available, mean imputation was used. Patient distance data were imputed by median within their municipality if missing.

Counts below five general practices was anonymised as<5. Statistical analyses were performed using Stata (version 18.0).

## Results

### Latent profile analysis

Throughout 2017–2021 the study included, respectively, 1707, 1668, 1626, 1606, and 1596 general practices, where 1286 practices contributed data in all 5 years. The number of single-handed practices decreased from 959 (56.2%) in 2017 to 835 (52.0%) in 2021. The mean number of consultations with each of their registered older patients decreased from 13.4 to 12.5, 11.0, 10.9, and 10.2, respectively, from 2017–2021.

Data were missing for age and sex for 0.6% for GPs and seniority for 1.1%. Distance data were missing for 1% of patients in 2017 and 10% in 2021.

**Figure 1.**
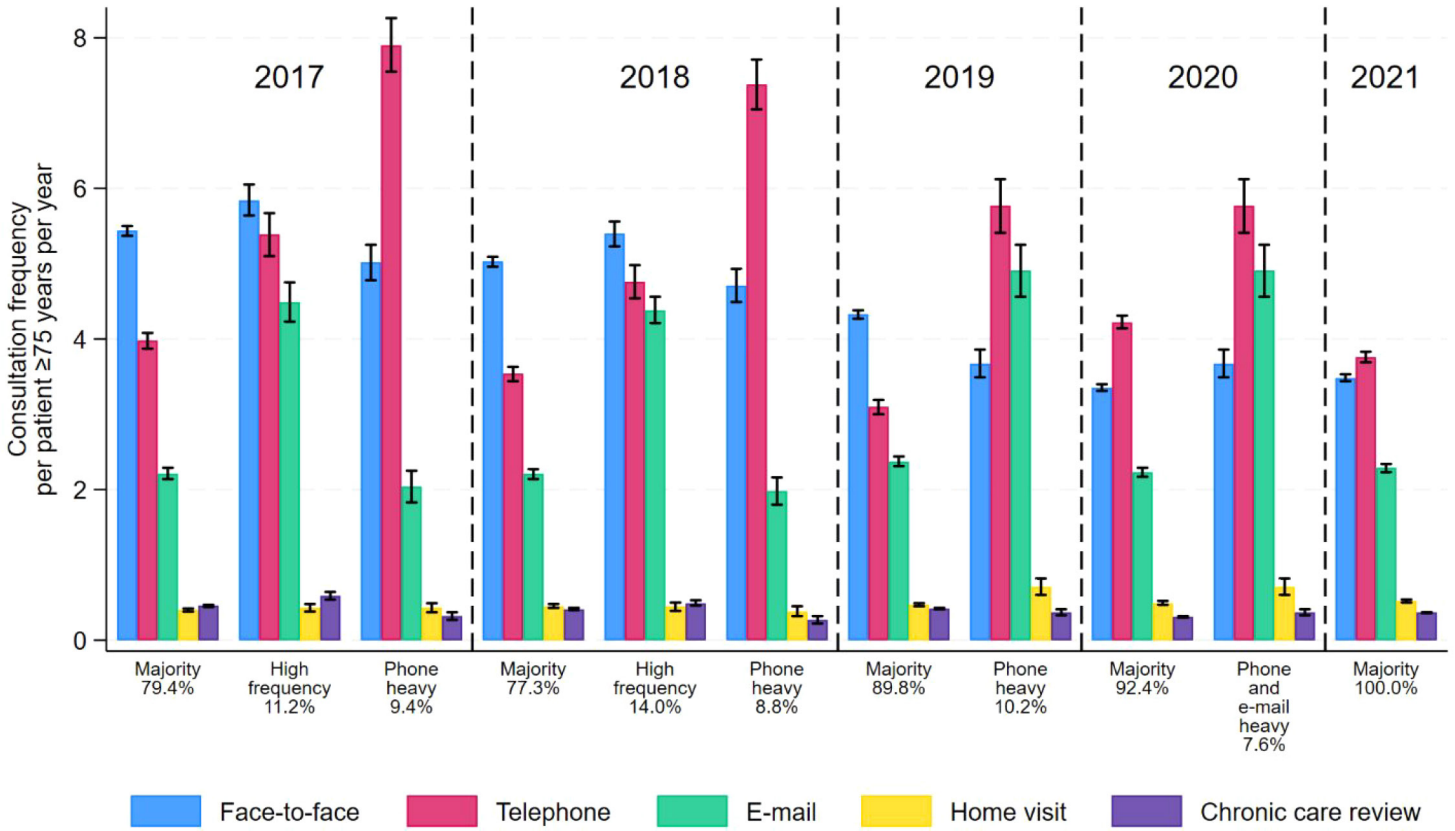
Latent profiles 2017–2021

All years included a ’Majority’ profile characterising 77%–90% of practices, which in 2017 exhibited a pattern of consultations in decreasing order of face-to-face, telephone, email, home visits, and chronic care reviews. Through the study period the profile provided increasingly fewer face-to-face consultations (2017: 5.4, 2021: 3.4) while the other consultation types were kept at similar levels as in 2017 (Figure 1, for exact numbers see Supplementary table S3).

A ’Phone heavy’ profile was found in 2017–2019 characterised by comprising 9%–10% of general practices and providing about the same level of consultations as the ’Majority’ profile except nearly double the telephone consultations.

**Figure 2.**
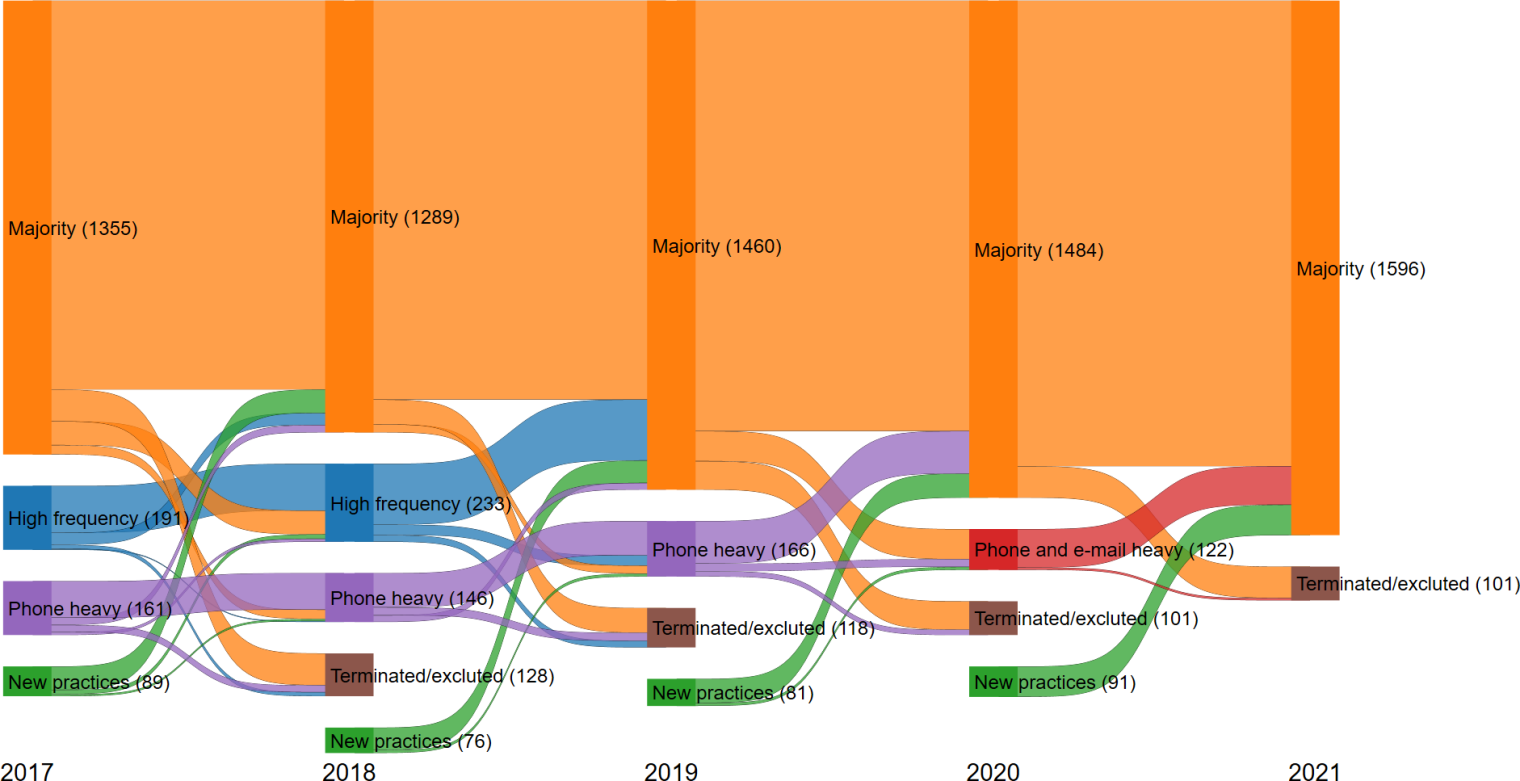
Movement between latent profiles 2017–2021. Numbers in parenthesis indicate the number of practices within each profile. Movements with fewer than 11 practices are anonymised and not shown in the figure. ’Terminated or excluded’ refers to practices that were terminated or excluded in the following year. ’New practices’ refers to newly started or included practices in the following year

A ’High frequency’ profile (12%–14% of practices) was found in 2017 and 2018, which provided higher levels of face-to-face and email consultations than the ’Majority’ and ’Phone heavy’ profiles, and more telephone consultations than the ’Majority’ profile. In 2019 most practices in the profile merged with the ’Majority’ profile (Figure 2).

In 2020, a minor ’Phone and email heavy’ profile (7%) used more face-to-face, telephone, and email consultations than the ’Majority’ and more email than face-to-face consultations. Most practices in this new profile came from the ’Majority’ profile in 2019.

### Profile stability over time

The overall average probability for profile membership was 95%, the minimum probability of profile membership was 35% for the ’High frequency’ profile in 2017, and the lowest proportion of practices with profile membership probability≥0.8 was 53% for the ’Phone and email heavy’ profile in 2020 (Supplementary table S4).

Figure 2 shows movements from year to year of general practices across the profiles. For practices starting in the ’Majority’ profile in 2017, 88% stayed through to 2021 (Supplementary table S5).

### Associations with practice factors

**Figure 3.**
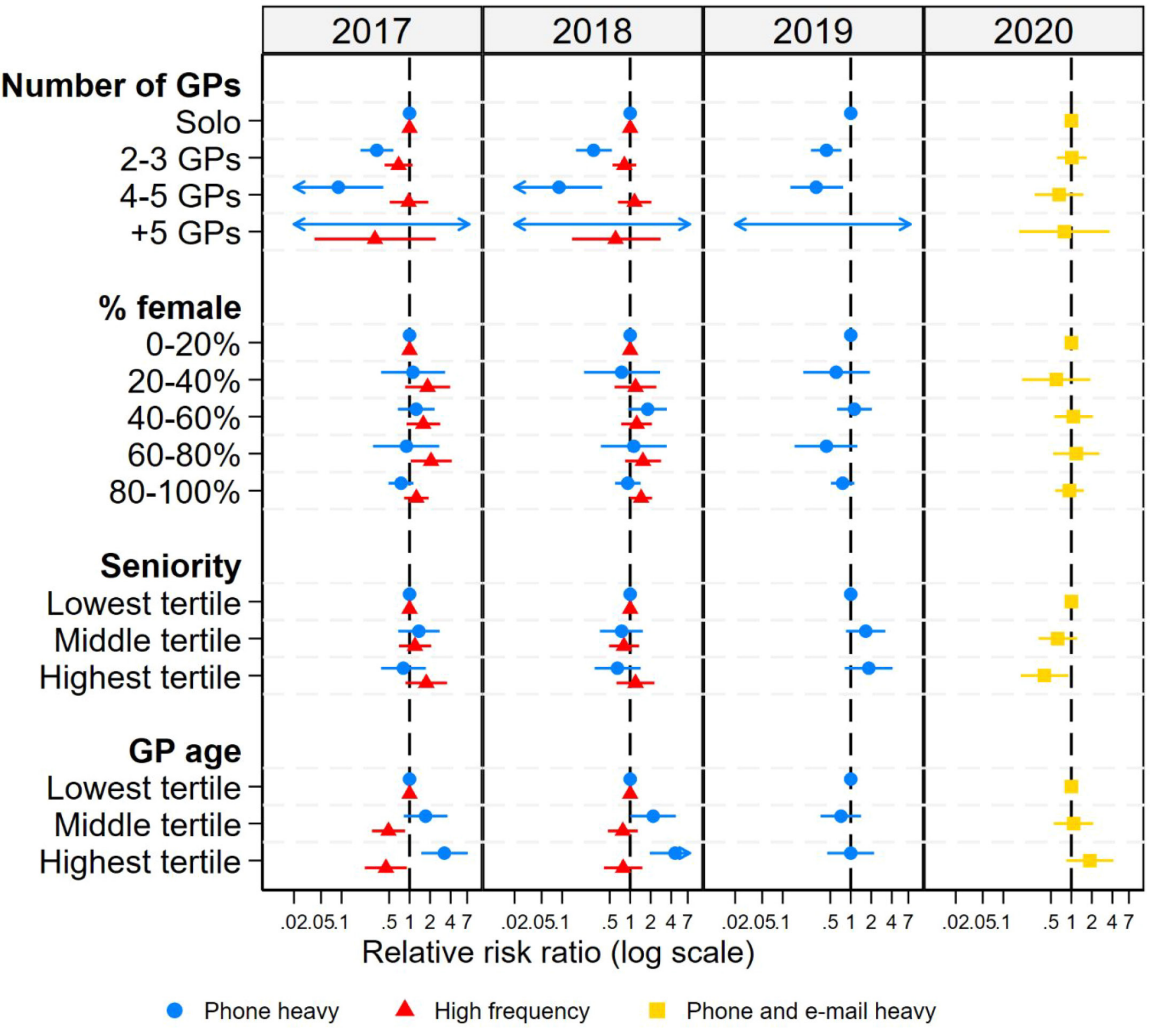
Multinomial logistic regression of latent profile membership and general practice factors 2017-–2020 (reference: the ’Majority’ profile). No regression could be performed for 2021 owing to only one latent profile found

’Phone heavy’ practices were more often single-handed practices and the GPs more often in the middle or highest age tertile compared with the ’Majority’ (Figure 3, for exact number see Supplementary table S6). No associations were found between latent profiles and the GPs’ sex. The ’Phone and email heavy’ profile in 2020 was associated with the GPs having fewest years of working experience (highest tertile of seniority relative risk ratio 0.40 [95% confidence interval {CI} = 0.18 to 0.90]). No other significant differences were found between the profiles throughout the years (Figure 3).

## Discussion

### Summary

The consultation patterns for older patients across general practices are rather uniform when adjusting for patient factors. From 2017–2021, the practices can be categorised into a ’Majority’ and three temporary profiles. The temporary profiles differ from the ’Majority’ profile mainly by providing more specific consultation types either by twice the telephone (’Phone heavy’, 2017–2019, associated with GPs being older and working single-handed), by face-to-face, telephone, and email (’High frequency’, 2017–2018), or by telephone and email (’Phone and email heavy’, 2020). During the study, the practices trended towards a more uniform pattern of consultations and overall fewer consultations with only one profile found in 2021.

### Strengths and limitations

The use of high-quality virtually complete nationwide registers reduced the risk of selection and information bias.

Our analysis of daytime consultations as a whole comprised of many different consultation types is a strength compared with the current literature focusing on a single consultation type.^2–4^ Additionally, our focus on groupings of general practices that do exist (latent profiles), and not theoretical or extreme cases improves the representativeness of our results.

The latent profile analysis is limited by trying to force general practices into a profile. If the true underlying distribution of latent profiles are comprised of many small but very distinct profiles our model will be limited by trying to form profiles with at least 5% of general practices. This might have been the case in 2021 where we found only one profile. Nonetheless, general practices trended towards the ’Majority’ profile throughout each year (Figure 2). Data from 2022 and onwards might have helped to strengthen or dismiss this trend. Unfortunately, data beyond 2021 was not available at the time of the study.

The frequency of consultations may be limited by market factors, where an ongoing GP shortage^13^ may mean the available resources in the general practices do not match the patients’ demand. This might have led to fewer consultations in practices more affected by the GP shortage; for example, rural areas.

### Comparison with existing literature

Our study of patterns of consultations with older patients in general practice shows a preference for face-to-face consultations with most general practices using this as the main consultation type. A Danish qualitative study during the COVID-19 pandemic showed that GPs in general have a preference for face-to-face consultations and that they planned to resume their usual practice when circumstances allowed them to do so.^14^ Preferences for consultation type is most likely also driven by patient preferences with older patients strongly preferring telephone to online consultations and slightly preferring face-to-face to remote consultations.^15^ GPs are also economically incentivised towards face-to-face above telephone consultations by a higher remuneration for the former (€21.54 [approximately 18.27 GBP] versus €4.20 [approximately 3.55 GBP] as of 2024).^16^

We found that 9%–10% of practices had a substantially higher use of telephone consultations than the ’Majority’. A possible reason may be workload issues. While the evidence is sparse on telephone as an alternative to face-to-face consultations, it has been shown to reduce time spent on consultations although requiring more follow-up than face-to-face consultations.^17^ Further, telephone consultations may more easily be delegated to practice staff (for example, nurses or secretaries) than face-to-face consultations. However, the ’Phone heavy’ profile was more often comprised of single-handed GPs and older GPs than the ’Majority’. A Swiss study found no association between practice type (single-handed or partnership) or GP age and crude consultation rates.^18^ To our knowledge no studies have investigated how GP age or practice type affects preference for telephone consultations for older patients or in general. Single-handed practices have been associated with higher continuity of care,^19^ patient reported access, and overall patient satisfaction,^20^ all of which may be possible through providing more overall and telephone consultations; however, none of the studies assessed this factor.

In 2020, we found a ’Phone and email heavy profile’. During the COVID-19 pandemic there were doubts as to whether all patients with negative COVID-19 test should actively be informed by their GP or only certain subgroups. This led to some general practices registering many email and telephone consultations, which may explain the origin for this new profile. The rules for remuneration were clarified during the year.

Overall, general practices trended towards a more uniform pattern of consultations. This differs from previous literature, which shows substantial variation in consultation frequencies across the total population.^21,22^ The smaller variation observed in the older population may be attributed to the prevalence of multiple chronic diseases, requiring regular monitoring and follow-up, thereby leading to a more uniform consultation pattern. Additionally, GPs are experiencing a generational change where older GPs, often working single-handed, are retiring or otherwise leaving practice^23^ and many younger GPs preferring working in partnership practices,^24^ resulting in fewer single-handed and more partnership practices (Supplementary table S6), potentially contributing to increased uniformity in consultation patterns across general practices.

We have found no previous studies describing or investigating the composition of consultation types in general practice for comparison.

### Implications for practice

The worldwide shift in demographics towards an older population may be the largest challenge for healthcare systems, especially for general practice, which already faces a shortage of GPs in many countries. Our study provides evidence that general practices trends towards a more uniform pattern of consultations for older patients, although some practices service their older patients markedly differently from the majority. The alternative patterns of consultations involve more services, which from an isolated time and cost perspective must be a disadvantage in a tax-funded healthcare sector. However, if the increased level of consultations has positive downstream effects, such as decreased need for hospital and out-of-hours services, it may be better to use allocated healthcare resources in daytime general practice than in secondary services.

## Data

All data are available form Statistics Denmark and The Danish Health Data Authority upon application.

## Patient and public involvement

A panel of older patients and next of kin assisted with hypothesis generation prior to analysis. A panel of general practitioners assisted with interpretation of the study results.
